# The SERRATE protein is involved in alternative splicing in *Arabidopsis thaliana*

**DOI:** 10.1093/nar/gkt894

**Published:** 2013-10-16

**Authors:** Katarzyna Dorota Raczynska, Agata Stepien, Daniel Kierzkowski, Malgorzata Kalak, Mateusz Bajczyk, Jim McNicol, Craig G. Simpson, Zofia Szweykowska-Kulinska, John W. S. Brown, Artur Jarmolowski

**Affiliations:** ^1^Department of Gene Expression, Institute of Molecular Biology and Biotechnology, Adam Mickiewicz University, Poznan, Poland, ^2^Department of Molecular and Cellular Biology, Institute of Molecular Biology and Biotechnology, Adam Mickiewicz University, Poznan, Poland, ^3^Max Planck Institute for Plant Breading Research, 50829, Germany, ^4^Biomathematics and Statistics Scotland (BioSS), James Hutton Institute, Dundee DD2 5DA, Scotland, UK, ^5^Cell and Molecular Sciences, James Hutton Institute, Dundee DD2 5DA, Scotland, UK and ^6^Division of Plant Sciences, University of Dundee at the James Hutton Institute, Dundee DD2 5DA, Scotland, UK

## Abstract

How alternative splicing (AS) is regulated in plants has not yet been elucidated. Previously, we have shown that the nuclear cap-binding protein complex (AtCBC) is involved in AS in *Arabidopsis thaliana.* Here we show that both subunits of AtCBC (AtCBP20 and AtCBP80) interact with SERRATE (AtSE), a protein involved in the microRNA biogenesis pathway. Moreover, using a high-resolution reverse transcriptase-polymerase chain reaction AS system we have found that AtSE influences AS in a similar way to the cap-binding complex (CBC), preferentially affecting selection of 5′ splice site of first introns. The AtSE protein acts in cooperation with AtCBC: many changes observed in the mutant lacking the correct SERRATE activity were common to those observed in the *cbp* mutants. Interestingly, significant changes in AS of some genes were also observed in other mutants of plant microRNA biogenesis pathway, *hyl1-2* and *dcl1-7*, but a majority of them did not correspond to the changes observed in the *se-1* mutant. Thus, the role of SERRATE in AS regulation is distinct from that of HYL1 and DCL1, and is similar to the regulation of AS in which CBC is involved.

## INTRODUCTION

Alternative splicing (AS) is a widespread process that generates more than one spliced mRNA isoform from the same gene. One of the major consequences of AS is to increase protein diversity by the inclusion or exclusion of peptide sequences or protein domains. The number of genes that undergo AS is ∼95% in human (1,2), and has recently increased to >60% of intron-containing genes in *Arabidopsis thaliana* (3,4). More than 75% of AS events occur within the coding sequence of the genes, and can generate proteins with new structures and biological functions (5–8). However, a significant number of AS events in coding regions generates premature termination codons, which potentially target transcripts for degradation by the nonsense-mediated decay (NMD) pathway. Thus, AS can also modulate gene expression through the production of mRNA isoforms, which are degraded by NMD (3,6,9–13). In both plants and animals, ∼20% of all AS events take place within untranslated regions: 5′ UTR (12–15%) or 3′ UTR (3–6%), which can affect transport and stability of mRNAs, create new initiation codons or polyadenylation sites, generate upstream open reading frames, trigger NMD or shift the reading frame (13–15).

AS events include alternative 5′ and 3′ splice site selection, intron retention, exon skipping and mutually exclusive exon splicing (5,16,17). In plants, intron retention is the most frequent alternative event (45–56%) (6,11,14,18,19) but appears to have much less impact at the transcript level (4). Alternative 3′ and 5′ splice sites account for ∼22 and 10% of events, respectively, and ∼4% have both 5′ and 3′ alternatively spliced sites. Only 8% of alternative events in plants involve exon skipping, in contrast to animals where exon skipping is the most common form of AS (58% of events) (6,15,19,20). AS of some genes in plants is evolutionarily conserved, suggesting its important role in plant development (21). The best-characterized example is that of serine/arginine (SR) protein splicing factor genes that undergo frequent AS. Moreover, SR proteins can regulate the AS of their own pre-mRNA, pre-mRNAs of other SR proteins and of target genes (22–28). With the exception of SR proteins, PTB and GRP7, little is known about proteins that regulate AS in plants (22–34). Previously we have shown that the plant nuclear cap-binding complex (CBC), consisting of two subunits (CBP20 and CBP80), can influence AS preferentially affecting AS of the first intron, and particularly at the 5′ splice site (35).

It has been shown that inactivation of either the *AtCBP80* or *AtCBP20* genes leads to pleiotropic developmental defects similar to the phenotype observed in Arabidopsis mutants of *SERRATE* (At*SE*) (36–39). SERRATE is a zinc finger protein that is mostly localized in nuclear Dicing-bodies (D-bodies), and plays a crucial role in microRNA (miRNA) biogenesis in plants. AtSE acts together with the endonuclease DICER-LIKE 1 (DCL1) and the double-stranded RNA-binding protein HYL1, in efficient and accurate processing of primary miRNAs (pri-miRNAs) to mature miRNAs (36,40–42). However, in *cbc* mutants, reduced miRNA levels and increased pri-miRNA levels were also observed (43–45), suggesting that both, AtSE and the CBC complex, have a role in miRNA biogenesis. Similarly, in the *se-1* mutant, accumulation of some partially spliced pre-mRNAs was also described, suggesting a role for AtSE in splicing of mRNAs (43). Interestingly, the loss of either AtCBC or AtSE activity often affected splicing of the first intron in a transcript (35,43).

In this article, using Bimolecular Fluorescence Complementation (BiFC), pull-down and co-immunoprecipitation experiments, we show that both subunits of AtCBC, AtCBP20 and AtCBP80 interact with AtSE. Moreover, we used the sensitive high-resolution reverse transcriptase-polymerase chain reaction (RT-PCR) AS panel (13,31,35,46) to analyze the effect of the *se-1* mutation on the AS profiles of 285 Arabidopsis genes. We have found that AtSE influences AS of a number of genes often affecting selection of 5′ splice site of first introns, similar to AtCBC, suggesting that the CBC and SERRATE cooperate in selection of alternative splice sites. Additionally, using RNA immunoprecipitation (RIP) we show that AtSE can directly bind selected target RNAs, confirming its role as a splicing regulator. We also found that changes observed in the *se-1* mutant did not correspond with the changes observed in Arabidopsis mutants of other key proteins that interact with AtSE, and are involved in plant miRNA biogenesis, *hyl1-2* and *dcl1-7*, suggesting that SERRATE has a function in regulation of AS in plants, which is distinct from its role in miRNA biogenesis.

## MATERIALS AND METHODS

### Plant material and growth conditions

*Arabidopsis thaliana* wild type and mutant lines in the Columbia (Col-0) ecotype were used for all analyses: the homozygous T-DNA insertion lines *hyl1-2* (SALK_064863) (47) and the point mutants *se-1* (48) and *dcl1-7* (49). Plants were grown in a growth chamber (SANYO MLR-350H) under controlled environmental parameters: humidity of 70%, temperature 22°C, 16 h light/8 h dark photoperiod regime at 150–200 μmol m^−^^2^s^−1^. Rosette leaves were harvested 35 days after sowing seeds, and frozen in liquid nitrogen. For each experiment, at least three biological replicates were harvested. Homozygous *dcl1-7* plants were identified using PCR. *Arabidopsis thaliana* (L.) Heynh. ecotype Columbia suspension-cultured T87 cells were grown in a growth chamber (Gallenkamp) with continuous illumination (100 μmol m^−2^s^−1^) at 22°C, with rotary shaking at 120 rpm in mJPL3 medium (50). The cultures were renewed weekly; 5 days after passaging T87 cells were used for protoplast preparation.

### Preparation of constructs for protein–protein interaction and subcellular localization studies

For co-localization, full-length (FL) AtCBP80, AtCBP20 and AtSE were amplified with gene-specific primers containing SalI and BamHI, or SalI and EcoRI restriction sites, and were then cloned into pSAT6-ECFP-C1 or pSAT4-EYFP-C1 (51), resulting in pSAT6-ECFP:AtSE, pSAT4-EYFP:AtCBP20 and pSAT4-EYFP:AtCBP80. For the BiFC analysis, PCR products were cloned into pSAT4-cEYFP-C1-B, pSAT4A-cEYFP-N1, pSAT1-nEYFP-C1 or pSAT1A-nEYFP-N1 (52), resulting in pSAT1-nEYFP-AtCBP20, pSAT1A-CBP20-nEYFP, pSAT4-cEYFP:AtCBP20, pSAT4A-AtCBP20:cEYFP, pSAT1-nEYFP:AtCBP80, pSAT1A-AtCBP80:nEYFP, pSAT1-nEYFP:AtSE, pSAT1A-AtSE:nEYFP, pSAT4-cEYFP:AtSE and pSAT4A-AtSE:cEYFP. For negative control experiments, free N-terminus of Enhanced Yellow Fluorescent Protein (nEYFP) and C-terminus of Enhanced Yellow Fluorescent Protein (cEYFP) fragments (from pSAT1-nEYFP-C1 and pSAT4-cEYFP-N1, respectively) in combination with complementary plasmids containing the protein sequences under study were used. To construct multicassette BiFC vectors, the expression cassette from pSAT6A-mRFP-N1 (52) was first cloned into the PI-PspI site of pPZP-RCS2 (53) to produce the pPZP-RCS2-mRFP vector. Afterward, expression cassettes from previously prepared pSAT vectors were transferred into the I-SceI and AscI sites of the pPZP-RCS2-mRFP vector to create pPZP-RCS2-nEYFP-cYFP:AtCBP20-mRFP and pPZP-RCS2-nEYFP:AtCBP80-cEYFP-mRFP. Sequences of inserts were confirmed for each construct. Sequences of primers used for construct preparation are listed in Supplementary Table S1.

### Protoplast transfection

The fusion constructs used for protein visualization and BiFC analyses were introduced into *A. thaliana* protoplasts prepared from suspension-cultured T87 cells or rosette leaves, as described previously (54–56). Protoplasts were analyzed for fluorescence 20–35 h after transfection using an epifluorescence microscope AxioObserver Z1 (Zeiss).

### Microscopy

Subcellular localization of fusion proteins was examined with a fluorescence microscope AxioObserver Z1 (Zeiss) equipped with a CCD camera AxioCam MRm (Zeiss) using a 63× air objective lens, or a confocal laser scanning microscope SP5 (Leica) using a 63× water objective lens. For the fluorescence microscope, specific filters for ECFP (excitation 436/20 nm, emission 480/40 nm) and Enhanced Cyan Fluorescent Protein (EYFP) (excitation 500/20 nm, emission 535/30 nm) were used. Excitation in the confocal was achieved with an Argon laser at 514 nm (EYFP), and with Helium-Neonium laser at 543 nm monomeric Red Fluorescent Protein (mRFP). Fluorescence was observed using the emission spectrum range of 523–560 nm (EYFP) and 571–635 nm (mRFP). Images were arranged using ADOBE PHOTOSHOP (Adobe Systems).

### Immunoprecipitation

Arabidopsis plants overexpressing AtSE:FLAG and AtHYL1:FLAG proteins were prepared in the *se-1* and *hyl1-2* mutant background, respectively. AtSE and AtHYL1 protein-coding sequence was amplified using AtSEfor, AtSErev, AtHYLfor and AtHYLrev primers (Supplementary Table S1). The products were cloned into the pEarlyGate202 plasmid, and transformed into *Agrobacterium tumefaciens AGL1*. Agrobacterium-mediated floral dip transformation was used to introduce the *FLAG-SERRATE* transgene into the *se-1* mutant genome, and the *FLAG-HYL1* transgene into the *hyl1-2* mutant genome. Homozygous transgenic plants had a restored wild-type phenotype and produced AtSE:FLAG or AtHYL1:FLAG proteins as confirmed by western blot (Figure 3A). After 35 days, leaves from control and transgenic plants were vacuum-infiltrated with 1% formaldehyde for 10 min, quenched with 125 mM glycine and frozen in liquid nitrogen. The nuclear proteins were extracted as follows: the frozen material was resuspended in Buffer I [0.4 M sucrose, 10 mM Tris–HCl, pH 8.0, 10 mM MgCl_2_, 0.035% β-mercaptoethanol (β-ME), one protease inhibitor tablet (Roche) per 50 ml of buffer], vortexed vigorously, filtered through Miracloth and centrifuged for 30 min at 3000*g* at 4°C. The pellet was resuspended in 1 ml of Buffer II [0.4 M sucrose, 10 mM Tris–HCl, pH 8.0, 10 mM MgCl_2_, 0.035% β-ME, 1% Triton X-100, protease inhibitor tablets (Roche)] and centrifuged for 10 min at 12 000*g* at 4°C; this step was repeated two to three times until a white pellet was visible. After the last centrifugation, the pellet was resuspended in 300 µl of Buffer II and loaded onto 900 µl of Buffer III [1.7 M sucrose, 10 mM Tris–HCl, pH 8.0, 2 mM MgCl_2_, 0.035% β-ME, 0.15% Triton X-100, protease inhibitor tablets (Roche)]. After 1 h of centrifugation at 16 000*g* at 4°C the pellet containing nuclei was collected and resuspended in lysis buffer [10% sucrose, 100 mM Tris–HCl, pH 7.5, 5 mM EDTA, 5 mM EGTA, 300 mM NaCl, 0.75% Triton X-100, 0.15% sodium dodecyl sulphate (SDS), 1 mM dithiothreitol (DTT), protease inhibitor tablets (Roche)]. After 1 h of shaking at 1000*g* at 4°C, the sample was centrifuged for 15 min at 14 000*g* at 4°C, and the supernatant containing nuclear protein lysate was collected. For co-immunoprecipitation experiments, anti-FLAG antibody-coupled magnetic beads (Sigma) were gently rotated overnight at 4° with the nuclear protein lysate, then washed four times with lysis buffer and eluted by boiling in sample buffer (50 mM Tris–HCl, pH 6.8, 10% glycerol, 2% SDS, 10 mM DTT, 0.1% bromophenol blue). Immunocomplexes were separated on 10% SDS-polyacrylamide gel electrophoresis (PAGE), transferred to polyvinylidene difluoride (PVDF) membrane (Millipore) and analyzed by western blot using anti-AtCBP20, anti-AtCBP80, anti-AtHYL1 (Agrisera AS09530, AS09531, AS06136) or anti-FLAG (Sigma) antibodies.

### Protein pull-down assay

AtSE FL coding sequence and core fragment (core, residues 194–543) were amplified using AtSEFLfor and AtSEFLrev, AtSEcorefor and AtSEcorerev primers, respectively (Supplementary Table S1), and cloned into the pMalc4e plasmid. The plasmids were used for subsequent transformation of *Escherichia coli* strain BL21(DE3)RIL. Overexpression of FL and core fragment of SERRATE fused with maltose binding protein (MBP) was performed as follows: 2 h after induction by 0.4 mM isopropyl β-D-1-thiogalactopyranoside (IPTG), cells were harvested and sonicated (15 cycles of 30 s ON and 30 s OFF; Bioruptor Plus, Diagenode) in MBP buffer [20 mM Tris–HCl, pH 7.4, 0.2 mM NaCl, 1 mM EDTA, protease inhibitor tablets (Roche)]. After sonication, lysates were centrifuged for 15 min at 14 000*g* at 4°C, and the supernatants containing protein extract were collected. The same protocol was carried out for MBP-GFP production. To obtain AtCBP20, AtCBP80 and the TPR domains of the SGT1 protein, *in vitro* translation in the presence of [^35^S]-methionine (HARTMANN ANALYTIC) was performed using TNT T7 Coupled Wheat Germ Extract System (Promega). For pull-down experiments, the MBP-AtSE FL, MBP-AtSE core and MBP-GFP were bound to the amylose resins (New England Biolabs), then washed three times with MBP buffer and incubated with labeled AtCBP20, AtCBP80 and TPR domains of SGT1 in phosphate buffer (28 mM NaH_2_PO_4_, 72 mM Na_2_HPO_4_, 250 mM KCl and 0.5% Triton X-100) for 2 h at 4°C. Next, the resins were washed four times with phosphate buffer, and protein complexes were eluted with 10 mM maltose. The labeled proteins were separated on 14% SDS-PAGE and detected with an image analyzer (FLA-5000, FUJIFILM).

### RNA immunoprecipitation

For RIP experiments, the nuclear protein extract was immunoprecipitated as described above. After washing of the beads, co-precipitated RNAs were eluted from IP samples with TRIZOL (Invitrogen). cDNA synthesis was carried out with an oligo (dT)_15_ primer using Superscript III reverse transcriptase (Invitrogen), according to the manufacturer’s protocol. Amplification was carried out in 10 µl reaction mix containing 5 µl of Power SYBR Green PCR Master Mix, 4 µl of 0.5 µM primers mix and 1 µl of template. The qPCR was performed for 40 cycles under the following cycling conditions: 95°C for 10 min, 40 cycles of 95°C for 15 s, 60°C for 1 min (Applied Biosystem 7900 HT thermocycler). Primers used for qPCR are listed in Supplementary Table S1.

### RNA isolation and high-resolution RT-PCR

Total RNA was isolated from 35-day-old rosette leaves using the RNeasy Plant Mini Kit (Qiagen). RNA was extracted from three biological repeat samples for each line. cDNA synthesis was carried out with an oligo (dT)_15_ primer using Superscript III reverse transcriptase (Invitrogen), according to the manufacturer’s protocol. The efficiency of cDNA synthesis was assessed by RT-PCR amplification of the ACT12 (At3g46520) cDNA fragment. After first-strand cDNA synthesis, 1 μl of the cDNA template per reaction were used for each PCR amplification with gene/AS-specific oligonucleotide primer pairs. Amplification was carried out in a 25 µl reaction mix containing 2.5 μl 10× PCR buffer with MgCl_2_ (Roche), 4 μl nucleotide mix (1.25 μM of each dNTP, Promega), 0.75 μl of combined primers (100 μM stock) and Taq DNA Polymerase (5U/μl, Roche). PCR was performed for 24 cycles under the following conditions: 94°C for 2 min, 24 cycles of 94°C for 15 s, 50°C for 30 s, 72°C for 1 min and completed with 10 min at 72°C. AS-specific primers were designed to amplify the expected alternatively spliced mRNA isoforms that were selected based on either published AS events or taken from five different Arabidopsis/plant bioinformatics databases: ASIP (http://www.plantgdb.org/ASIP/EnterDB.php), TIGR (http://www.tigr.org/tdb/e2k1/ath1/), RIKEN (http://rarge.gsc.riken.jp/a_splicing/index.pl), ASTRA (http://alterna.cbrc.jp/) and TAIR 7.0 (http://www.arabidopsis.org/index.jsp) (46). The size of RT-PCR products ranged between 60 and 700 bp. To visualize the RT-PCR products on an ABI3730 capillary sequencing machine (Applied Biosystems), each forward primer was labeled with 6-carboxyfluoresceine. Splicing and statistical analysis were performed as described previously (35). To validate statistical significance of RIP results, the *t*-Student’s test was used, and in the analyses of AS comparisons, the hypergeometric test was used (31). In both cases, *P* < 0.05 was applied for the validation.

## RESULTS

### SERRATE interacts with both subunits of AtCBC in the cell nucleus

To analyze the subcellular localization of the *A. thaliana* cap-binding protein complex, AtCBC, and the SERRATE protein, AtSE, the two subunits of the nuclear CBC, AtCBP20 and AtCBP80, were fused with enhanced yellow fluorescent protein (EYFP), and AtSE was fused with enhanced cyan fluorescent protein (ECFP) at the N-termini for all proteins studied. The constructs were transiently expressed in *A. thaliana* protoplasts. As shown in Supplementary Figure S1 (top panel), AtCBP20 co-localized with AtSE in the nucleus. Co-localization between AtCBP80 and AtSE in the nucleus was also detected, but AtCBP80 was also present in the cytoplasm of transfected protoplasts (Supplementary Figure S1, bottom panel). The cytoplasmic localization of AtCBP80 can be explained by relatively low level of endogenous AtCBP20 in transfected protoplasts, as the AtCBP20 is necessary for import of AtCBP80 from the cytoplasm to the nucleus, as shown by us previously (55). Taken together, our results indicated that both components of AtCBC co-localize with AtSE in the cell nucleus.

**Figure 1.**
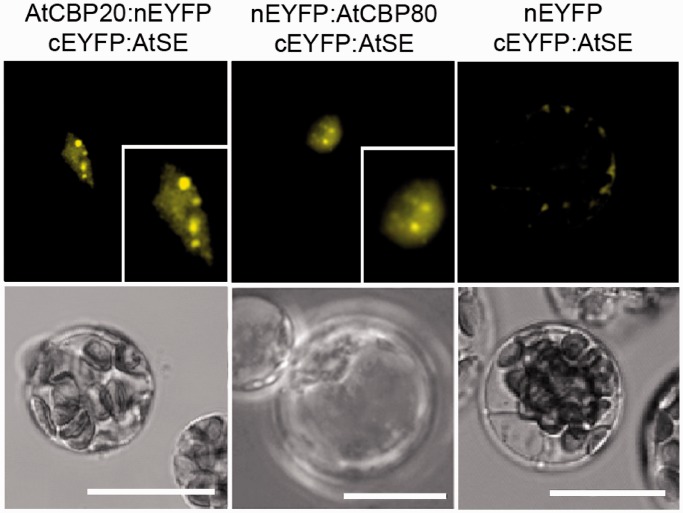
BiFC analysis of the interaction between AtCBC subunits and AtSE. *Arabidopsis thaliana* protoplasts were co-transfected with combinations of different plasmids encoding EYFP or cEYFP fused to AtSE, AtCBP20 and AtCBP80 coding sequences. Insets represent a magnified view of the representative nucleus for each interaction. The lower panel shows transmission images of the transfected protoplasts in which fluorescence was observed (upper panel). Scale bars = 20 µm.

Next, we used BiFC to directly study the physical interaction between the proteins of the nuclear CBC and AtSE in living plant cells. FL AtCBP20, AtCBP80 and AtSE were fused to complementary nonfluorescent regions of EYFP (52), and used for protoplast co-transfection (Supplementary Table S2). As a positive control for the BiFC experiment, we used AtCBP20 and AtCBP80 fused to complementary parts of EYFP, and observed a strong nuclear BiFC signal (Supplementary Table S2) confirming the interaction previously shown by Fluorescence Resonance Energy Transfer (FRET) between the two components of AtCBC (55). As a negative control, we used plasmids containing EYFP fragments fused with N-terminus or C-terminus of AtSE, AtCBP20 or AtCBP80 proteins in combination with free complementary EYFP fragments (Figure 1, right panel; Supplementary Table S2). However, in some protoplasts, a weak fluorescence signal was detected all over the transfected protoplast. These interactions were most likely nonspecific owing to unusually high expression levels of recombinant proteins.

**Figure 2.**
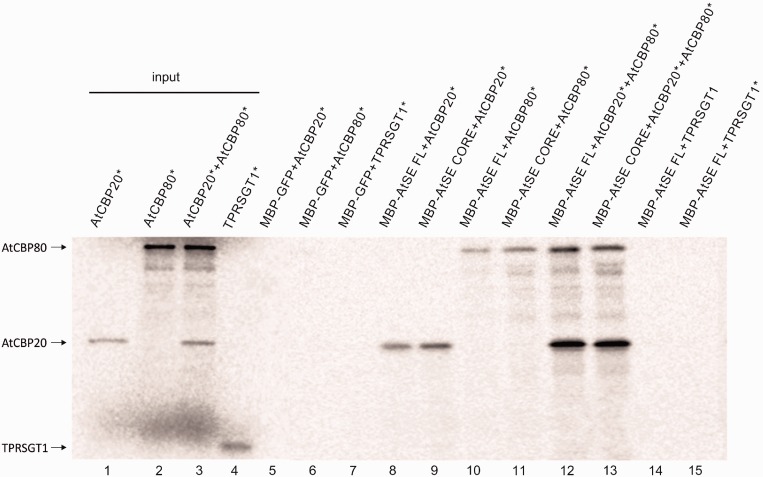
The interaction between FL AtSE or its core fragment (residues 194–543; AtSE core), and AtCBP20 and/or AtCBP80. AtSE FL, AtSE core and GFP proteins were overexpressed in bacteria in fusion with MBP; AtCBP20, AtCBP80 and TPRSGT1 (used as a negative control) were synthesized in the presence of [^35^S]-methionine (an asterisk in the protein name abbreviation means that the protein was labeled). The complexes were selected on amylose beads, separated on 14% SDS-PAGE and detected by exposure to an image analyzer. Inputs represent one-twentieth of the samples used in the experiment.

Strong fluorescence of reconstituted EYFP was observed in protoplasts co-transfected with combinations of BiFC vectors containing sequences of AtSE and AtCBP80 or AtSE and AtCBP20 (Figure 1, left and middle panels). In both cases, the fluorescence in most cell nuclei was not homogenous, and several brighter spots were observed in the nucleoplasm of 90% nuclei of cells co-transfected with AtCBP20 and AtSE, and in 70% of the nuclei transfected with plasmids coding AtCBP80 and AtSE (Supplementary Figure S2 and Supplementary Table S2). The observation that AtSE binds both AtCBP20 and AtCBP80 are in contrast to the results recently published by Wang *et al.* (2013) (57), which did not detect an interaction between AtSE and the larger subunit of CBC either in BiFC or in pull-down experiments. Although we showed the interaction between AtSE and both AtCBC subunits (above), this interaction was not seen in all conformations of plasmids used in BiFC (see Supplementary Table S2). For example, the AtSE fused at the C terminus with the C-terminal fragment of EYFP did not give a signal when co-transfected with AtCBP80 fused with N-terminal fragment of EYFP. Therefore, we confirmed our observation with independent methods: protein pull-downs and co-immunoprecipitation. For the protein pull-down assays (Figure 2), we analyzed the interactions between [^35^S]-methionine-labeled AtCBP20, AtCBP80 and the TPR domains of the SGT1 protein with full length (FL) or the core domain of AtSE fused with the MBP. No interactions were observed when negative controls (TPR domains of the SGT1 or MBP-fused GFP) were added to the sample (Figure 2, lane 5–7, 14, 15). However, we detected a signal confirming the interaction with AtSE when AtCBP20 and/or AtCBP80 were added to the sample either separately or together (Figure 2, lane 8–11 and 12, 13, respectively). Both CBP subunits bind to the FL AtSE protein as well as to the AtSE core; the core fragment of AtSE acts as a protein-binding platform (58). Interestingly, the binding of the AtCBP20 with AtSE seems to be stronger than AtCBP80 with AtSE, and the strongest interaction was observed when AtCBP20 and AtCBP80 interact with AtSE in a complex (Figure 2, line 12, 13). The stronger signal shown by AtCBP20 may be the result of a higher efficiency pull-down, as a result of better folding of the smaller subunit of AtCBC.

As mentioned above, Wang *et al.* (2013) (57) did not observe the interaction between AtSE and atCBP80 in pull-down experiments. However, as they have not shown that the recombinant AtCBP80 protein is able to bind AtCBP20, we suggest their negative result might come from incorrectly folded AtCBP80 protein used in their analyses.

**Figure 3.**
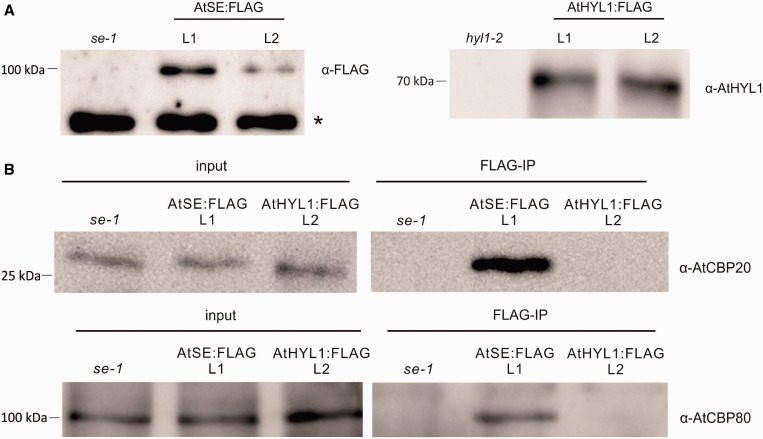
The interaction between AtSE and AtCBC. (**A**) Western blot analysis using anti-FLAG and anti-AtHYL1 antibodies confirmed the presence of AtSE:FLAG (left panel) and AtHYL1:FLAG (right panel) in two different lines of transformed *se-1* (L1 and L2) and *hyl1-2* (L1 and L2) mutant plants, respectively. (**B**) AtCB20 (top panel) and AtCBP80 (bottom panel) were detected by western in complexes co-immunoprecipitated with anti-FLAG antibodies from transgenic plants expressing AtSE:FLAG, but not from plants expressing AtHYL1:FLAG. Transgenic lines L1 and L2 were used in the case of plants expressing AtSE:FLAG and AtHYL1:FLAG, respectively. The position of the closest protein marker is indicated on the left; an asterisk marks an unidentified protein cross-reacting with anti-FLAG antibodies.

We also performed a co-immunoprecipitation experiment with the nuclear protein lysate extracted from plants overexpressing either AtSE:FLAG or AtHYL1:FLAG proteins (Figure 3B). The latter was used as a negative control: according to our previous observation, the AtHYL1 protein does not interact directly with AtCBP20 and AtCBP80 (55). Western blot analyses with anti-AtCBP80 and anti-AtCBP20 antibodies revealed the presence of both proteins in the fraction co-immunoprecipitated with SERRATE but not with HYL1 (Figure 3B), confirming the AtCBC/AtSE interaction in plant cells. Similar to the pull-down experiments, in the western blot performed after co-immunoprecipitation, we did observe stronger signal from AtCBP20 then AtCBP80, although both CBC subunits were detected in the immunoprecipitates analyzed (Figure 3B, bottom panel). Taken together, the BiFC analyses performed in protoplasts as well as pull-down and immunoprecipitation experiments indicated that in *A. thaliana* both AtCBC subunits form a complex with AtSE. Moreover, the AtCBC/AtSE complex seems to be localized exclusively in the nucleus where it is dispersed within the nucleoplasm or accumulated in several distinct subnuclear regions.

### AS is affected in the Arabidopsis *se-1* mutant

The subunits of the *A. thaliana* nuclear cap-binding protein complex, AtCBP20 and AtCBP80, as well as the SERRATE protein are involved in both miRNA biogenesis and pre-mRNA splicing (36,40–45). We have recently shown that the nuclear cap-binding protein complex (AtCBC) is also directly involved in AS of some Arabidopsis genes, and in most cases the AtCBC influences 5′ splice site selection of first introns (35). As the AtSE participates in pre-mRNA splicing and interacts with AtCBC (Figures 1–3), we asked whether SERRATE is also involved in the regulation of AS in plants. To answer this question, we used the high-resolution RT-PCR AS panel (46) containing a set of primers designed to examine 302 AS events in 285 Arabidopsis genes. These genes encode mainly transcription factors, splicing factors and stress-related proteins (for the full list see 35). The panel included a range of different types of AS events: alternative 5′ or 3′ splice site selection, alternative position (5′ and 3′ splice sites altered in the same splicing event), exon skipping and intron retention. Splicing profiles were determined for wild-type Col-0 and the *se-1* mutant, and the ratio of the alternatively spliced products for each gene was compared. Means and standard errors were calculated for three separate independent experiments.

**Figure 4.**
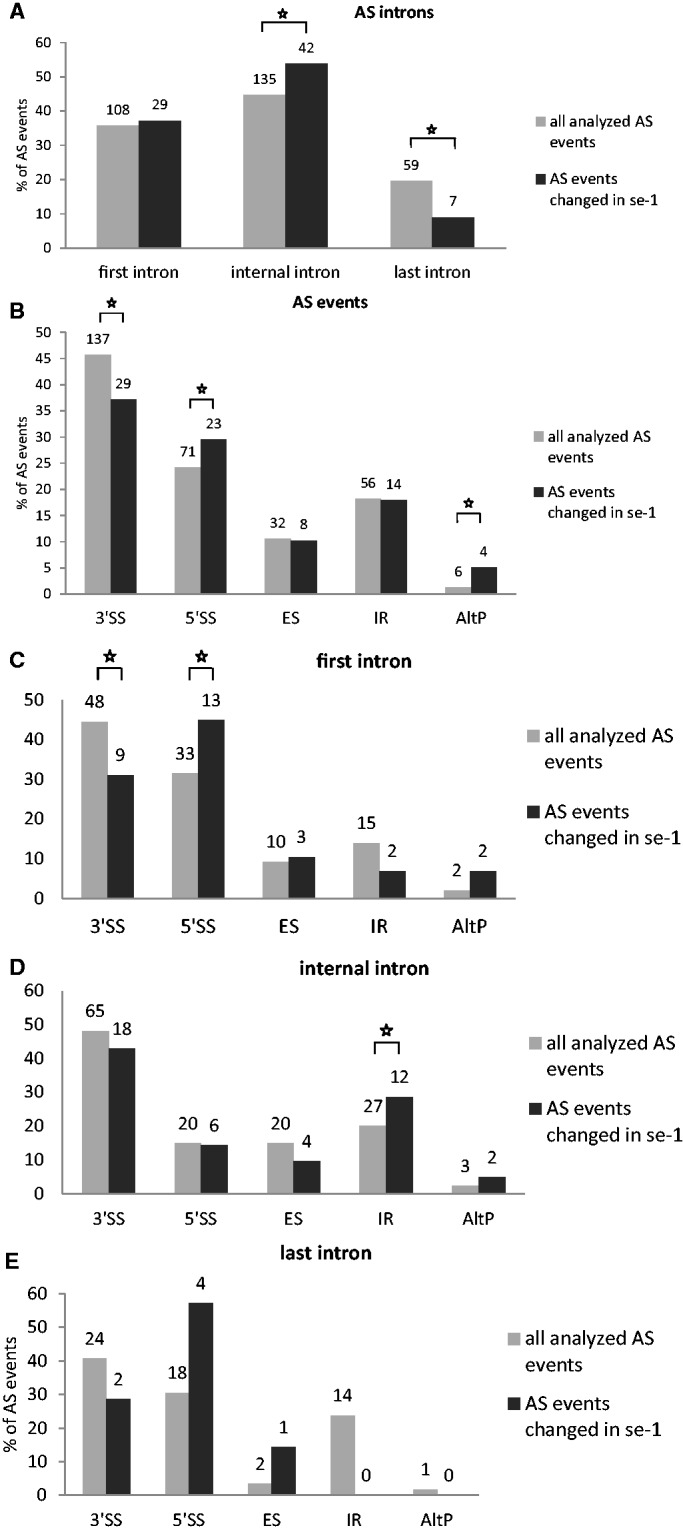
Distribution of the AS events presented for the total AS events (302 events/285 genes, gray bars), and those that changed in the *se-1* mutant (78 events/67 genes, black bars). (**A**) Distribution of the position of the alternatively spliced introns (first intron, internal intron, last intron); (**B**) Distribution of the alternatively spliced events: alternative 3′ splice site (3′SS), alternative 5′ splice site (5′SS), exon skipping (ES), intron retention (IR), alternative 3′ and 5′ splice sites (AltP); (**C**) Distribution of the AS events within first introns, internal introns and last introns. Numbers above bars indicate the number of alternatively spliced events. Statistical significance was tested using the hypergeometric test; an asterisk marks significant changes (*P* < 0.05).

Significant changes (>3%; *P* < 0.10) in the ratios of AS isoforms in the *se-1* mutant, in comparison with the wild-type plant, were found in 78 AS events (in 67 genes) (Table 1). To identify whether introns or AS events in particular positions in the transcripts were influenced preferentially by the AtSE protein, we compared the genes with significantly changed AS profiles in the *se-1* mutant to all analyzed genes from the high-resolution RT-PCR AS panel. In *se-1*, the changes involved mainly AS events located within internal introns (42 events, 54%) and first introns (29 events, 37%), with only seven cases of an AS event located within the last introns (7 events, 9%) (Figure 4A). Similarly, of the 302 AS events on the panel, 108 (36%) events were in the first intron, 135 (45%) events were in internal introns and 59 (19%) events were in the last intron (Figure 4A). In the *se-1* mutant, the significantly changed AS events included mostly changes in alternative 3′ or 5′ splice sites (29 and 23 events, 37 and 29%, respectively), but also included intron retention (14 events, 18%) and exon skipping (8 events, 10%) (Figure 4B). In contrast, alternative 3′ and 5′ splice sites among all of the AS events/genes on the RT-PCR panel accounted for 46 and 24% of the total, respectively (Figure 4B). This reflects the fact that AS in plants occurs more frequently at alternative acceptor sites (3′ splice sites) than in 5′ donor sites (22 versus 10%) (6,15). Thus, in the *se-1* mutant, there was an increased number of genes with significant changes at alternative 5′ splice sites than in the overall AS events analyzed. When we looked at the distribution of different AS events within first and internal introns, we found that in the *se-1* mutant AS within first introns was mostly affected at the 5′ splice site (45%), while AS within internal introns was mostly affected at the 3′ splice site (43%) (Figure 4C). Thus, AtSE preferentially influences AS of the first intron of a pre-mRNA at alternative 5′ splice sites.

**Table 1.** Significant changes in AS isoform abundance in the *se-1* and *cbp* mutants

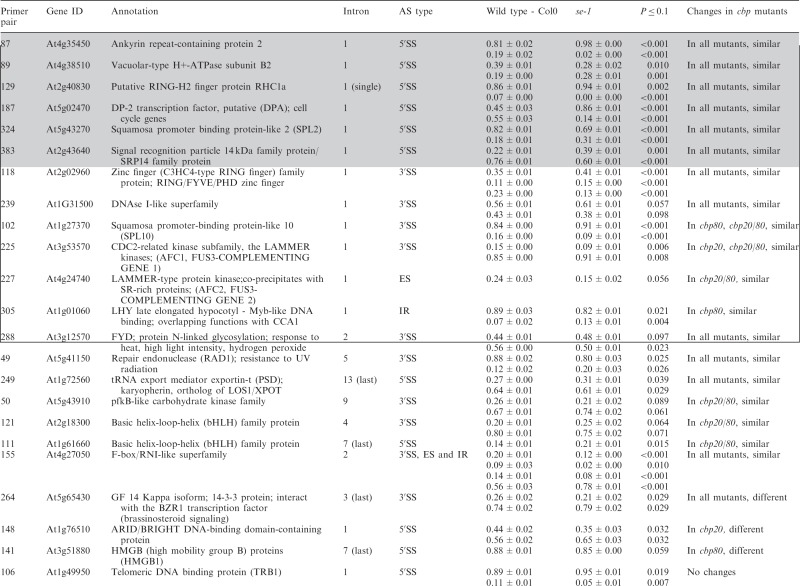
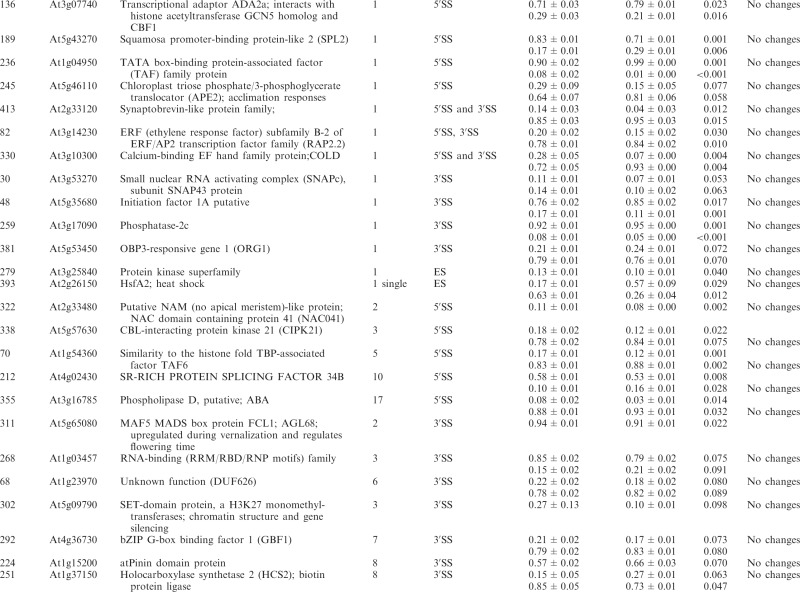
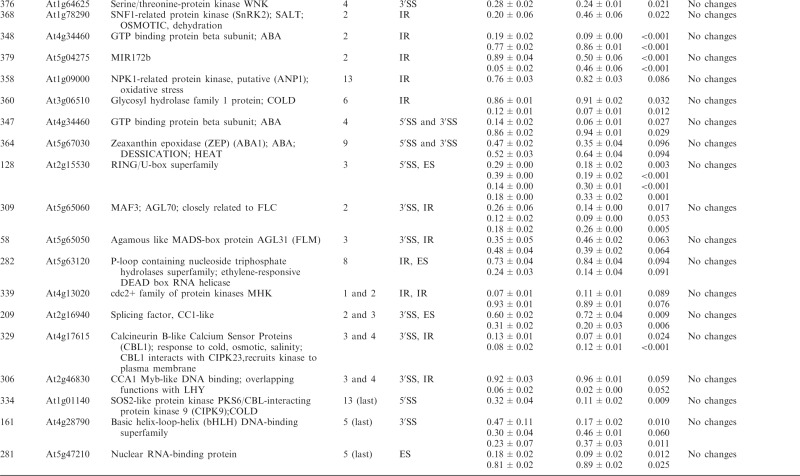

Significant changes in AS isoform abundance in the *se-1* mutant and all *cbp* mutants within first introns are boxed, significant changes in the *se-1* mutant and all *cbp* mutants at the 5′ splice site within first introns are shadowed by gray. In the column ‘changes in cbp mutants’, significant changes that are similar in both *cbp* mutants and the *se-1* mutant were indicated as ‘similar’, whereas changes that do not correlate were indicated as ‘different’. The table contains only isoforms that changed significantly (>3%; *P* ≤ 0.1).

**Figure 5.**
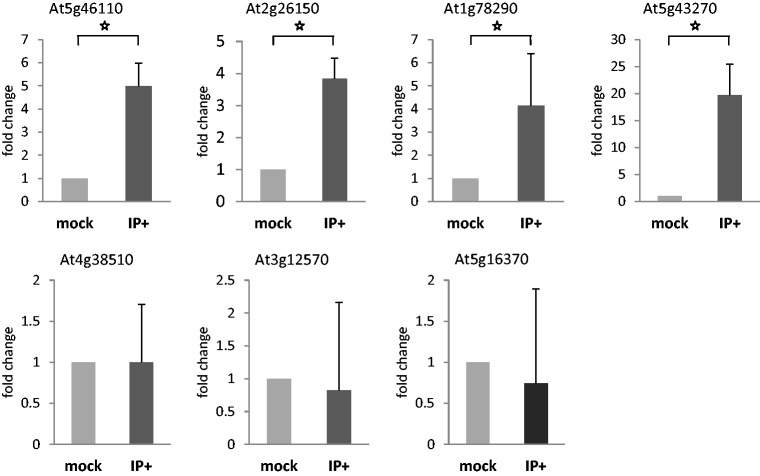
Interactions of AtSE with selected mRNA targets detected by RIP. Immunoprecipitation followed by RNA isolation and RT-qPCR confirmed *in vivo* interactions of AtSE:FLAG with candidate gene transcripts whose AS profile was changed in the *se-1* mutant; intron-less mRNA was used as a negative control (At5g16370). The level of transcripts co-precipitated from transgenic plant expressing AtSE:FLAG (IP+) or wild type plants (mock) using anti-FLAG antibodies were normalized to the inputs. Means ± SD are presented based on three biological replicates; statistical significance was tested using the *t*-Student’s test; an asterisk marks significant changes (*P* < 0.05).

To confirm the regulatory role of AtSE in AS of Arabidopsis gene transcripts, we checked whether AtSE can directly bind selected target transcripts. Using the transgenic plants overexpressing the FLAG-tagged AtSE protein, we performed immunoprecipitation using anti-FLAG antibodies followed by subsequent isolation of bound RNAs and reverse transcription. qPCR was performed on six arbitrarily chosen genes whose AS profile was changed in the *se-1* mutant (Supplementary Table S1). The results revealed that AtSE co-immunoprecipitates four of six transcripts analysed, suggesting that SERRATE can directly bind those mRNAs (Figure 5). No binding was observed in the case of one intron-less mRNA (At5g16370) used in this experiment as a negative control. Thus, the role of SERRATE as a regulator of AS of selected gene transcripts in *A. thaliana* was confirmed.

### AtSE and AtCBC cooperate in regulation of AS of some genes

The preference of the AtSE protein in affecting AS at alternative 5′ splice sites within first introns resembles our previous observations of the *cbp20*, *cbp80(abh1)* and *cbp20/80* double mutants (35). As AtCBP20 and AtCBP80 both interact with AtSE, we asked whether the AS profiles observed in the *se-1* mutant were similar to those observed previously in the *cbp* mutants. Of the 67 genes with significant changes in AS profile in the *se-1* mutant, 22 also had significant changes in AS in the *cbp* mutants (35) (Table 1).

Of the 22 AS events, which were affected in both the *se-1* and *cbp* mutants, 19 showed the same direction of AS changes (Table 1). The majority of these genes showed significant AS changes in the *se-1* and the double *cbp20/cbp80* mutant (18 genes), and 12 of these showed significant differences in the *se-1* and all three *cbp* mutants (Table 1). Two genes (primer pair 102: At1g27370, and 225: At3g53570) showed the same direction of AS splicing in the *se-1* mutant, the double *cbp20/cbp80* mutant and *cbp80(abh1)* or *cbp20*, respectively. One gene (305; At1g01060) showed the same direction in the *se-1* and *cbp80(abh1)* mutants. It is important to note that in the *cbp80(abh1)* mutant the level of AtCBP20 is extremely low, and is therefore to all extents and purposes similar to the double *cbp20/cbp80* mutant (55). Furthermore, among the 19 genes with common changes in the *se-1* and *cbp* mutants, the AS events were located for the most part within the first intron (12 of 19) (Table 1, boxed). Of these 12, six of the AS events were alternative 5′ splice sites (Table 1, shaded gray). Thus, almost a third (19/67) and a fifth (19/101) (35) of the significantly changed AS events in the *se-1* and *cbp* mutants, respectively, were common, and showed a similar behavior in AS, suggesting that both AtSE and AtCBP80/CBC are required for AS of the genes studied. As we showed previously for AtCBC, we did not observe any preference for selection of either cap-proximal or cap-distal alternative 5′ splice sites within the first introns. On the other hand, we did observe some influence on intron retention AS events (Table 1 and Figure 4B and C). However, we did not detect in our panel any significant influence on the levels of unspliced transcripts that might reflect an effect on general splicing efficiency in the *se-1* mutant. In addition, 45 genes (54 AS events) showed significant changes in AS profiles in the *se-1* mutant exclusively. For these genes, we did not observe any enrichment of AS events within first introns (16 events in first introns versus 38 events in internal and last introns) (Table 1). Thus, neither AtCBC nor AtSE seems to influence the general efficiency of splicing, but both factors participate in AS of some genes. The interaction between AtCBP80 and AtSE along with the preferential effects on the first intron and alternative 5′ splice site suggests that at least for some transcripts there is mutual cooperation of AtSE with the nuclear CBC in determining splice site choice. However, these factors also affect the AS of different subsets of genes and therefore can act independently in AS regulation.

### AS is also affected by other proteins involved in plant miRNA biogenesis

AtSE along with CBPs affect AS of a subset of genes putatively reflecting the interaction between SE and the cap binding complex (Figures 1–3), and recruitment of other splicing factors. Why and how SERRATE, which is mainly involved in miRNA biogenesis, affects AS of other genes independently is unknown. We therefore examined AS in mutants of other miRNA processing pathway factors that are known to interact with SE: HYL1 and DCL1. The *hyl1*-2 mutant is a T-DNA insertion mutant with no production of the HYL1 protein (47), and *dcl1-7* has a point mutation in the *DCL1* gene encoding the endonuclease directly involved in miRNA biogenesis; the *dcl1-7* mutant was used in the studies since the T-DNA inactivation of the *DCL1* gene is lethal (49). To address the question of whether mutations in *DCL1* and *HYL1* genes affected AS, and whether the effects resembled those of the *se-1* mutant, we again used the high-resolution RT-PCR panel to analyze AS events in *hyl1-2* and *dcl1-7* mutants.

**Figure 6.**
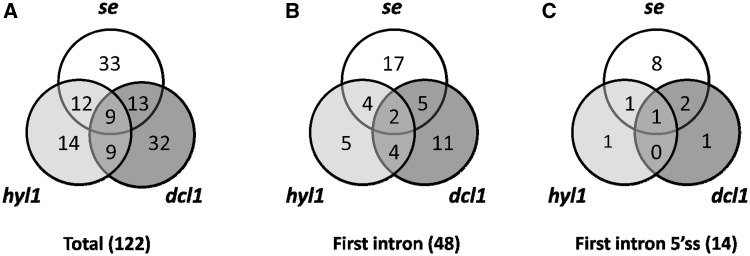
Distribution of AS events with significant changes in AS profiles in the *se-1*, *hyl1-2* and *dcl1-7* mutants (**A**) in total, (**B**) within first introns, (**C**) within first introns at the 5′ splice sites.

Of the 285 analyzed genes, we found significant changes in the ratios of AS isoforms in 122 genes in the *se-1*, *hyl1-2* and *dcl1-7* mutants, in comparison with wild type plants (Table 2). Of these 122 genes, 33 were observed exclusively in the *se-1* mutant, and 32 and 14 genes showed significant AS changes only in the *dcl1-7* or *hyl1-2* mutant, respectively. Interestingly, nine genes showed significant changes in AS in all three miRNA biogenesis mutants tested, while nine genes had changes in the *hyl1-2* and *dcl1-7* mutants, 12 in the *hyl1-2* and *se-1* mutants and 13 in the *se-1* and *dcl1-7* mutants (Figure 6A and Table 2).

**Table 2.** Significant changes in AS isoform abundance in the *se-1, hyl1-2* and *dcl1-7* mutants

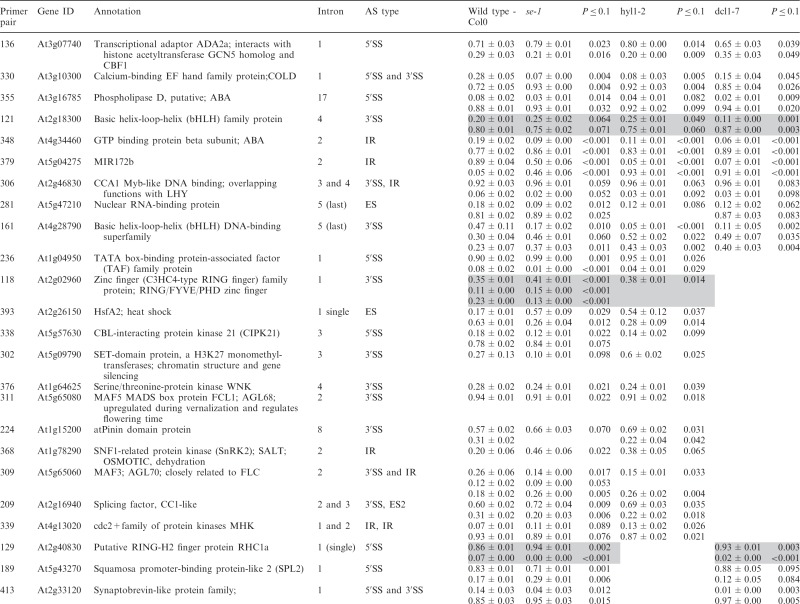
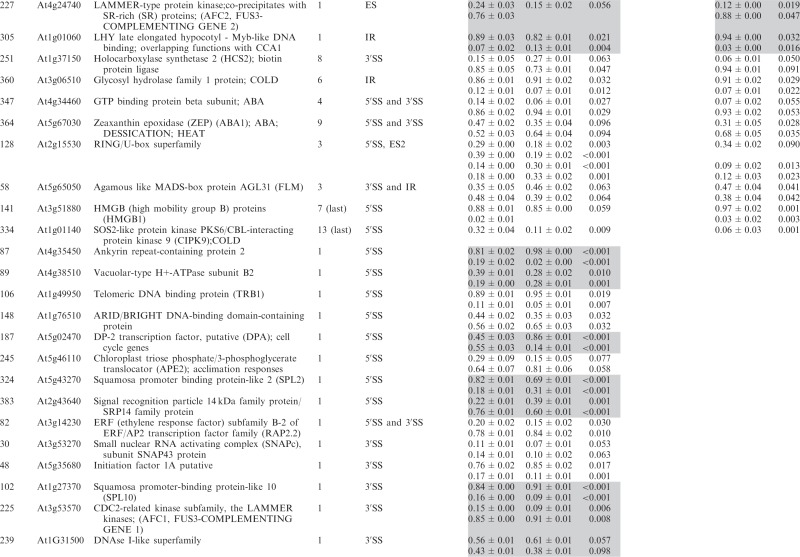
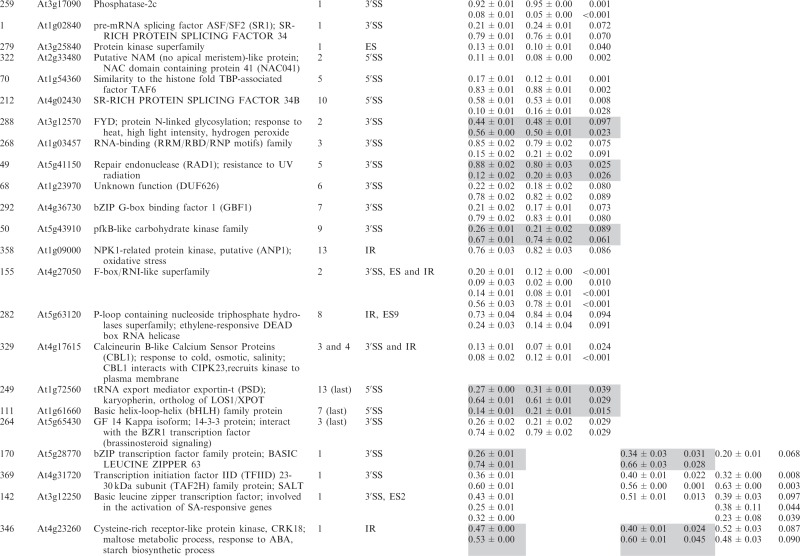
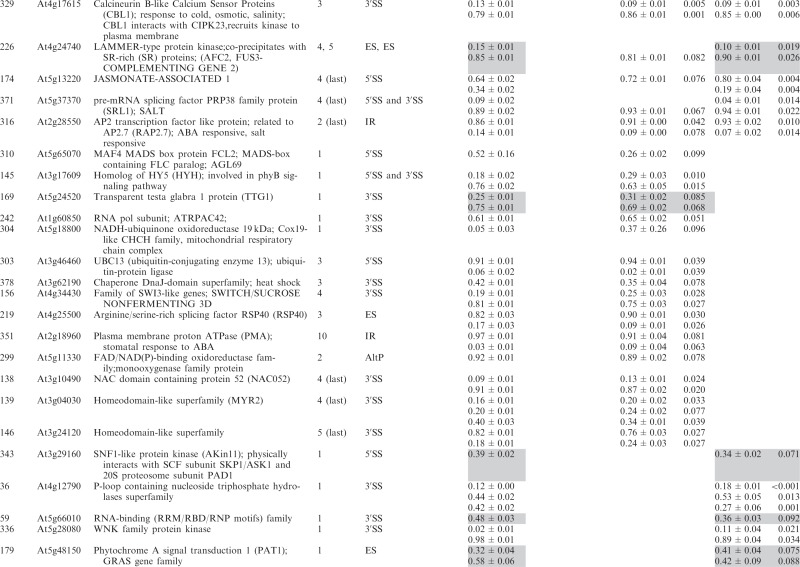
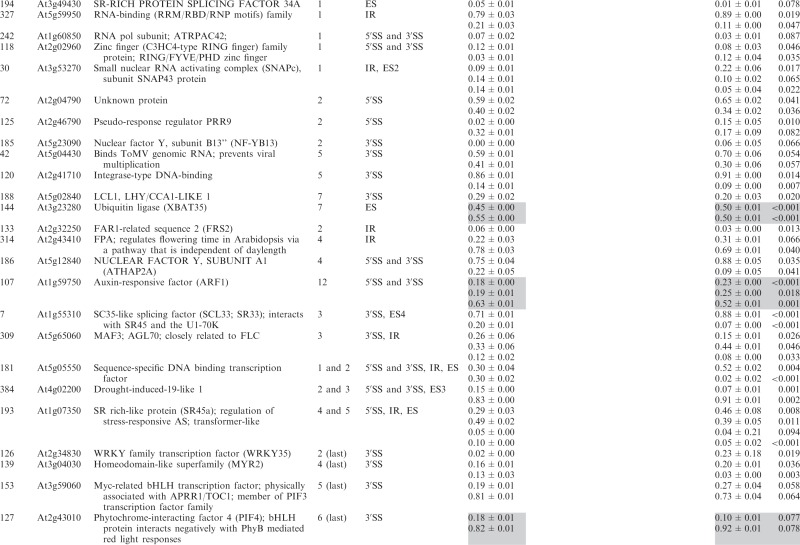
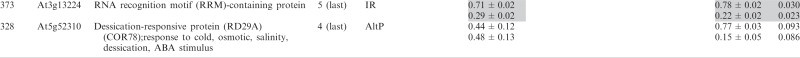

Significant changes in AS isoform abundance in the *se-1*, *hyl1-2*, *dcl1-7* mutants, which are also observed in *cbp* mutants (32) are shadowed by gray. The table contains only isoforms that changed significantly (>3%; *P* ≤ 0.1).

**Figure 7.**
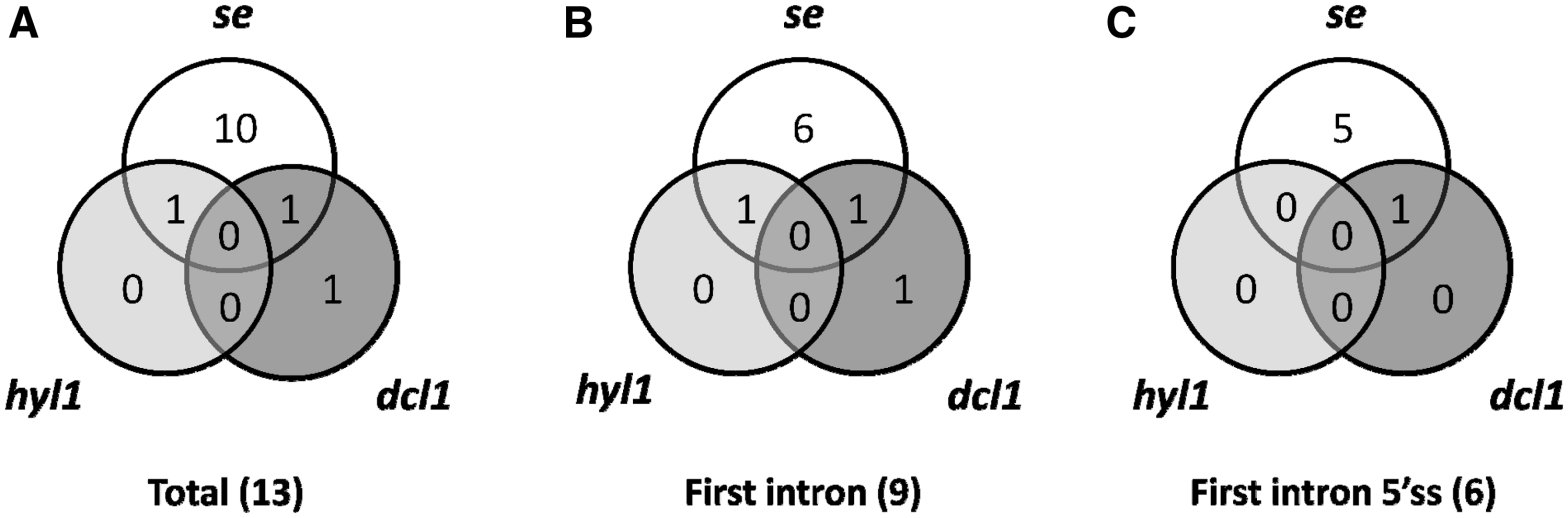
Distribution of AS events with significant changes in AS profiles in *se-1*, *hyl1-2*, *dcl1-7* compared with the all *cbp* mutants (**A**) in total; (**B**) within first introns; (**C**) within first introns at the 5′ splice sites.

Of the 33 genes affected only in the *se-1* mutant, 17 AS events were located within the first intron, and eight of them affected alternative 5′ splice sites (Figure 6B and C). Moreover, 13 of the 33 genes with significant AS changes observed in the *se-1* mutant also occurred in the *cbp* mutants, confirming our previous conclusion that the nuclear CBC and the SERRATE protein cooperate in selection of 5′ splice sites of some pre-mRNA first introns (Table 2, shaded gray, Figure 7). In the other miRNA biogenesis mutants we did not observe an enrichment of AS changes in introns located closest to the cap. This is illustrated by analyzing the 122 genes with AS changes in either *se-1*, *dcl1-7* or *hyl1-2*. Although 48 of these genes had significant AS changes in the first intron (Figure 6B and Table 2), only 14 affected selection of alternative 5′ splice sites (Figure 6C and Table 2). Twelve of these involved the *se-1* mutant with eight only in the *se-1* mutant, and two common to the *se-1* and *dcl1-7* mutants. Thus, the predominant effect of AS on alternative 5′ splice site selection at the first intron seen in the *cbp* and *se-1* mutants is not observed for the AS events affected in *dcl1-7* or *hyl1-2*. As a result, SE and CBC, which interact and associate with the cap structure, have clearly distinct effects on AS from DCL1 and HYL1.

## DISCUSSION

### AtSE is a novel factor involved in AS regulation

Previously we have shown that the plant nuclear CBC, consisting of two subunits, AtCBP20 and AtCBP80, influences AS, preferentially affecting AS of the first intron at the 5′ splice site (35), and this has since been also demonstrated in human cells (59). Here, we introduce another plant AS factor, SERRATE (AtSE), that acts in a similar way to CBC, mostly affecting selection of alternative 5′ splice sites within the intron that is the closest to the cap structure. The role of AtSE as a plant splicing factor was suggested previously when some unspliced intron-retaining pre-mRNAs were observed in *A. thaliana se-1, cbp80(abh1)* and *cbp20* mutants by microarray analysis (43). Significantly, in the few identified cases, splicing of the first intron seemed to be most sensitive to loss of either AtCBC or AtSE activity, suggesting that both CBC and AtSE influence splicing of plant pre-mRNAs in a similar manner (43). In this study, we have shown that AtSE affects AS, and that in many cases (28%) the changes of AS observed in the *se-1* mutant were similar to the changes observed previously in the *cbp* mutants. This suggests that for at least some genes, AS can be regulated coordinately by the nuclear CBC and AtSE. Such coordination could reflect a direct interaction between AtCBC and AtSE; we have shown here that both components of AtCBC co-localize with AtSE in the nucleus, and interestingly, both AtCBC subunits, AtCBP20 and AtCBP80, interact with AtSE *in vitro* and *in planta*. A similar interaction has been seen in human where the nuclear CBC interacts with the human SERRATE homolog—Ars2 (Arsenic Resistance Protein 2). In this case, immunoprecipitation of FLAG-Ars2 confirmed that Ars2 co-precipitates both the 80 and 20 kDa subunits of the human CBC (60). Additionally, AtSE was also identified in nuclear speckles in which SR splicing factors were enriched (61), and Ars2 was identified as a component of RNA–protein complexes enriched for spliceosomes (60), again potentially reflecting a role in splicing/AS.

Besides the AS changes common to both the *se-1* and *cbp* mutants, AtSE influenced AS of other genes. Previously, unspliced or pre-mRNAs with retained introns were observed for the *se-1* and *cbp* mutants (43). While this might suggest a general effect on splicing efficiency, it more likely reflects the resolution of microarrays, and we show that loss of AtCBC and AtSE does not affect splicing/AS of all introns but rather both factors preferentially participate in the selection of the 5′ splice site of the first intron.

### Involvement of AtSE and AtCBC in miRNA biogenesis

The main function of AtSE in plants is in the miRNA biogenesis pathway (36,40,41). Owing to its significant role in this process, a null mutation of the *SE* gene is lethal (40), and nonlethal mutants (e.g. *se-1*) lead to pleiotropic developmental defects with increased cauline leaf number, serrated leaf morphology and hypersensitivity to abscisic acid (ABA) (36,37). A similar but less severe phenotype is observed in Arabidopsis mutants of CBP20 and/or CBP80(ABH1) (37–39), suggesting that the role of both CBC subunits in plant miRNA biogenesis (43–45) is not as critical as that of AtSE, since the lack of both CBC subunits has only limited effects on the mutant phenotype (35,62–64). The CBC is thought to bind the capped pri-miRNA transcripts, and facilitate the loading of the miRNA processing machinery onto pri-miRNAs, analogous to its role in recruiting the splicing commitment complex onto pre-mRNAs (43–45). AtSE is thought to connect the CBC and miRNA processing machinery as it binds both AtCBC (Figures 1–3) and DCL1 or HYL1 as has been previously demonstrated (41,65). AtCBP80(ABH1) and AtSE also work together in splicing-mediated suppression of RNA silencing in Arabidopsis (66,67) again demonstrating the collaboration between these two proteins in RNA processing pathways. In human cells, Ars2 (the human homologue of SERRATE) and CBP80 co-precipitate with Drosha, and the depletion of CBP20 and CBP80, and of Ars2, results in similar defects in miRNA formation and miRNA-mediated silencing (60). In *Drosophila*, Ars2 and CBC are also required for miRNA function, Ars2 and Dicer-2 interact and Ars2 is involved in processing of dsRNAs into siRNA by Dicer-2 (68). As this process occurs in the cytoplasm, it suggests that the activity of Ars2 is not restricted only to the nucleus (68). Taken together, in several species, the SERRATE homologues appear to function as a bridging factor that co-transcriptionally binds CBC that is associated with the 5′ end of a pri-miRNA transcript, and recruits miRNA processing components to the substrate, hence increasing both the efficiency and precision of miRNA processing.

### AS in the mutants of miRNA biogenesis

The *se-1* mutant affected AS of a number of genes including a subset also affected by the CBC. The effect of AtSE on splicing can be explained by its interaction with the CBC on pre-mRNAs or, if it links the CBC to splicing factors/spliceosomal proteins, it is possible that it can also interact with such proteins independently of the cap to influence splicing. There are a number of other potential mechanisms by which AS could be affected in the *se-1* mutant through indirect effects of disruption of the plant miRNA biogenesis pathway. For example, a miRNA could target and degrade specific alternatively spliced transcript isoforms as has already been reported (69), and reduced production of the miRNA would affect the relative levels of isoforms. Alternatively, AS can affect the production of intronic miRNAs (70). We therefore also examined AS in the *hyl1-2* and *dcl1-7* mutants in addition to *se-1.* Surprisingly, all three mutants showed altered AS in a number of genes, although only nine genes had similar changes in AS profile common to all three mutants. These may reflect changes due to disruption of miRNAs, but so far only two of the genes are known targets of miRNAs. However, if plant miRNAs have a wider target range or off target effects than currently known, this might explain the impact on AS. The three mutants also affected AS of some genes uniquely. HYL1 is crucial for processing of only a subset of pre-miRNAs (47,71), which may explain the specific effect on particular genes. Disrupted interactions in the SE-HYL1-DCL1 complex may also affect miRNA biogenesis. For example, the *hyl1-2* mutant is a null T-DNA insertion mutant of HYL1 (71) but an amino acid substitution in the ATPase/DExH-box RNA helicase domain of DCL1 confers restoration of miRNA expression in the *hyl1-2* mutant background, implying that HYL1 may not be even required for miRNA processing (72). The interaction with HYL1 triggers structural rearrangements of DCL1 that activates its RNase III domain normally repressed by the helicase domain, similarly to human Dicer that is autoinhibited by its helicase domain, but can be activated by interaction with TRBP2 (73,74). Thus, in the *hyl1-2* mutant, the activity of DCL1 can be altered potentially affecting levels of some miRNAs. AtDCL1 is also involved in production of some siRNAs, which could also affect AS transcript isoform levels via degradation of targets or through methylation of DNA causing altered rates of transcription and subsequent changes in splice site selection (75). The effects of the *se-1*, *hyl1-2* and *dcl1-7* mutants on AS may also be due to altered transcript levels of genes encoding proteins involved in transcription, splicing or transport. Recently, HYL1 was shown to interact by its double-stranded RNA-binding (DRB) domain with other secondary structured RNAs, like transposons, recognizing structured RNA fragments, especially those with imperfect stem-and-loop structures (76). This raises the further possibility that HYL1 can also bind to regions of pre-mRNAs that form secondary structures, which could influence the recognition of acceptor or donor sites by splicing factors, resulting in alteration of constitutive splicing. On the other hand, in the absence of HYL1, DCL1 can associate with other DRB proteins, and is also capable of cleaving RNA hairpin structures, although in such cases the DCL1 endonuclease predominantly processes the substrate incorrectly (77). This could lead to production of incorrect small RNA molecules that could erroneously target other mRNAs/alternative isoforms for cleavage.

In summary, we observed changes in AS of transcripts in mutants of the miRNA biogenesis pathway, and while some of those affected in *se-1* can be explained owing to its interaction with the CBC, the reasons for the AS effects of the *hyl1* and *dcl1* mutants are not clear, and systematic experiments are needed to address this intriguing question.

## SUPPLEMENTARY DATA

Supplementary Data are available at NAR Online.

## FUNDING

Polish National Science Center [UMO-2011/01/M/NZ2/01435 to A.J. UMO-2012/05/N/NZ2/00880 to A.S. and UMO-2012/04/NZ2/00127 to Z.S.-K.]; the Polish Ministry of Science and Higher Education [4631/B/PO1/2010/39 to M.K.]; Biotechnology and Biological Sciences Research Council (BBSRC) [BB/G024979/1 - European Research Area network (ERA-NET) Plant Genomics (Plant Alternative Splicing and Abiotic Stress)]; Scottish Government Rural and Environment Science and Analytical Services division (RESAS). Funding for open access charge: Polish National Science Center [grant UMO-2011/01/M/NZ2/01435]; Adam Mickiewicz University, Faculty of Biology, Poznan, Poland.

*Conflict of interest statement*. None declared.

## Supplementary Material

Supplementary Data
