# Supplementary material for: The SERRATE protein is involved in alternative splicing in Arabidopsis thaliana
Source: Nucleic Acids Res. 2013 Oct 16;42(2):1224–44. doi: 10.1093/nar/gkt894 (PMC3902902; doi:10.1093/nar/gkt894)
Supplement: Supplementary Data [file supp_42_2_1224__index.html]

The SERRATE protein is involved in alternative splicing in Arabidopsis thaliana — The SERRATE protein is involved in alternative splicing in Arabidopsis thaliana — Supplementary Data 

# The SERRATE protein is involved in alternative splicing in *Arabidopsis thaliana*

## Supplementary Data

files

**Files in this Data Supplement:**

- Supplementary Data - pdf file
