# Supplementary material for: The SERRATE protein is involved in alternative splicing in Arabidopsis thaliana
Source: Nucleic Acids Res. 2013 Oct 16;42(2):1224–44. doi: 10.1093/nar/gkt894 (PMC3902902; doi:10.1093/nar/gkt894)
Supplement: Supplementary Data [file supp_gkt894_nar-03424-a-2012-File011.pdf]

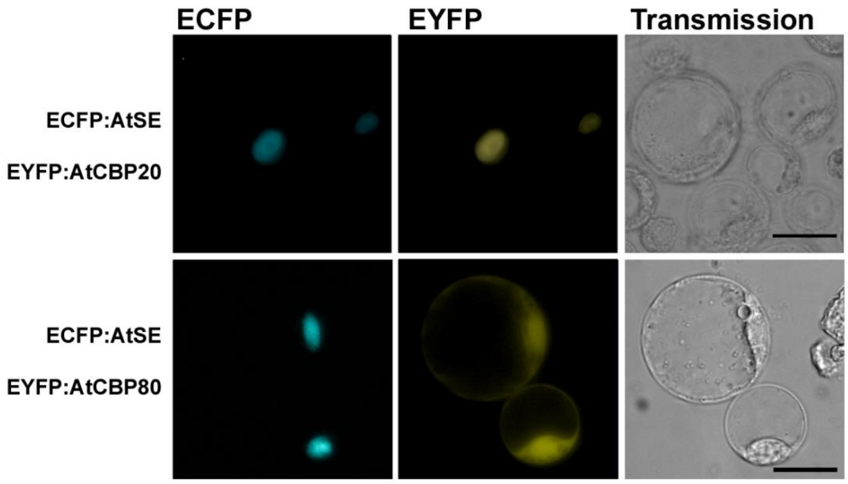

Figure S1. The subcellular localization of AtSE, AtCBP20 and AtCBP80 fusion proteins. Transfected *Arabidopsis thaliana* protoplasts were analysed by fluorescence microscopy. Scale bars: 20 um.

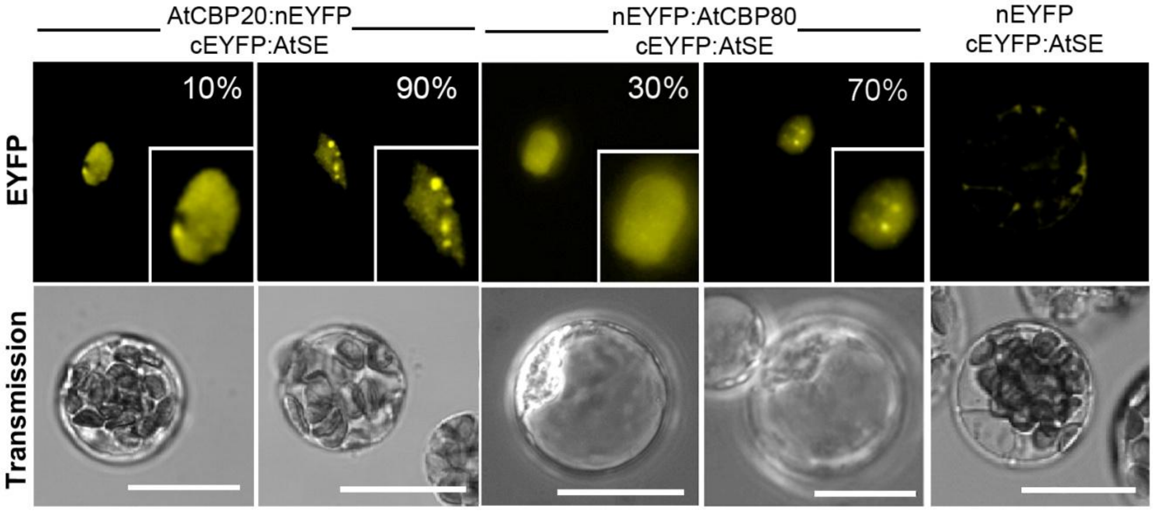

Figure S2. Bimolecular Fluorescence Complementation (BiFC) analysis of the interaction between AtCBC subunits and AtSE. *Arabidopsis thaliana* protoplasts were co-transfected with combinations of different plasmids encoding nEYFP or cEYFP fused to AtSE, AtCBP20 and AtCBP80 coding sequences. Insets represent a magnified view of representative nucleus for each interaction. Percent of nuclei with homogenous or speckled EYFP signals is presented in images. Scale bars = 20 µm.

Table S1. Primers used in subcellular localization studies, IP, pull-down and qPCR experiments

**Gene**	**Primers sequences**
Primer pairs used in subcellular localization studies	
AtCBP20	5’-AGGGTCGACATGGCTTCTTTGTTCAAGGAGC-3’<br>5’-CGGATCCTAGATCTTCTCTTCCGATCATCTTC-3’<br>5’-CGGATCCTTAAGATCTTCTCTTCCGATC-3’
AtCBP80	5’-CGGGTCGACATGAGCAATTGGAAAACTCTTC-3’<br>5’-CGGATCCTATGTAATGGGAATTGAAGTGCAG-3’<br>5’-CGGATCCTTAATGTAATGGGAATTGAAGTGC-3’
AtSE	5’-GGAATTCGATGGCCGATGTTAATCTTCCTCC-3’<br>5’-GCCGTCGACACAAGCTCCTGTAATCAATAAC-3’<br>5’-GCCGTCGACCTACAAGCTCCTGTAATCAAT-3’
Primers used in immunoprecipitation experiment	
AtSEfor	5’-CACCATGGCCGATGTTAATCTTCC-3’
AtSErev	5’-CTACAAGCTCCTGTAATCAATAACG-3’
AtHYLfor	5’-CACCATGGCCGATGTTAATCTTCC-3’
AtHYLrev	5’-CTACAAGCTCCTGTAATCAATAACG-3’
Primers used in pull-down experiment	
AtSEFLfor	5’-TCCGCTCGAGATGGCCGATGTTAATCTTCCTC-3’
AtSEFLrev	5’-ACGCGTCGACTTATTACTACAAGCTCCTGTAATCAATAACG-3’
AtSEcorefor	5’-TCCGCTCGAGGGATTGATGTCATACAAACAATTTATC-3’
AtSEcorerev	5’-ACGCGTCGACTTATTAATAGTTCTGAAAATACAGTTCTTCCCG-3’
Primer pairs used in qPCR	
At5g46110	5’- CATAAACAAGTTCTCCGATGG-3’<br>5’- CTCGAAAACAATGGTTGATGG-3’
At2g26150	5’- GGTTCGGAACCTTAACTATAC-3’<br>5’- ACAAACCACACTCAATGACTC-3’
At1g78290	5’- CATTGGAAGTAGCAGTATGGA-3’<br>5’- ATGTGTAGTAGTATCAGCAGG-3’
At5g43270	5’- CAATTCAGTTTCCGGCTATTG-3’<br>5’- TATCACTAGCTTGCTTGATCC-3’
At4g38510	5’- CATAGTTTCTACTGCCTTGGT-3’<br>5’- AGAATAATCAACCTGGCTTGC-3’
At3g12570	5’- TCCGGAAGGGTGAAAGTGAC-3’<br>5’- GATGAATGGCTCACATGCTG-3’
At5g16370	5’-TGTCCTTTGTGGATGAGCTG-3’<br>5’-CAACCTCGTCGTACCCATCT-3’

All restriction sites used in vector construction are underlined.

Table S2. Combination of vectors used for BiFC experiments.

**Plasmids used in experiments**	**EYFP fluorescence**
pSAT1-nEYFP:AtCBP20<br><br>and<br><br>pSAT4-cEYFP:AtSE	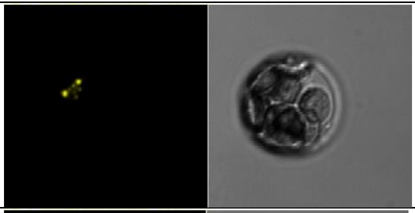
pSAT1-nEYFP:AtCBP20<br><br>and<br><br>pSAT4A-AtSE:cEYFP	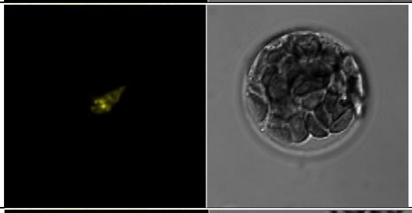
pSAT1A-<br>AtCBP20:nEYFP<br><br>and<br><br>pSAT4-cEYFP:AtSE	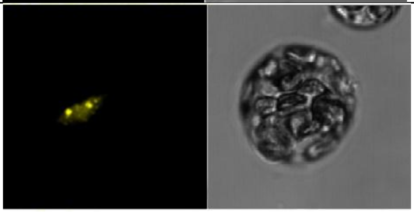
pSAT1A-<br>AtCBP20:nEYFP<br><br>and<br><br>pSAT4A-AtSE:cEYFP	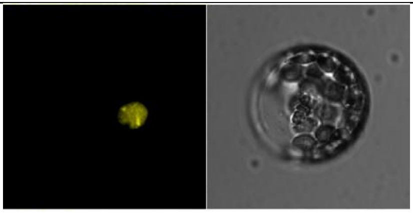
pSAT4-cEYFP:AtCBP20<br><br>and<br><br>pSAT1A-AtSE:nEYFP	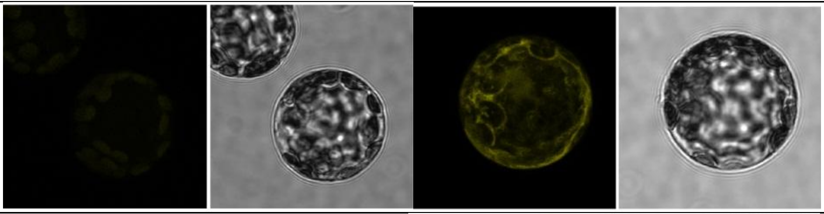
pSAT4-cEYFP:AtCBP20<br><br>and<br><br>pSAT1- nEYFP:AtSE	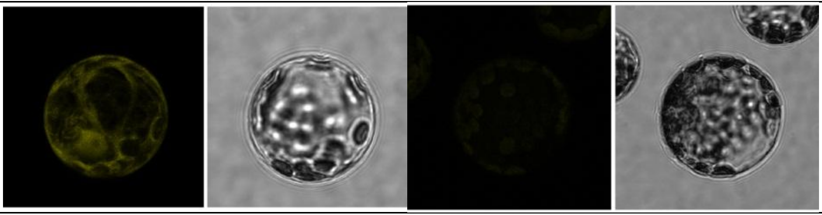
pSAT4A-<br>AtCBP20:cEYFP<br><br>and<br><br>pSAT1A-AtSE:nEYFP	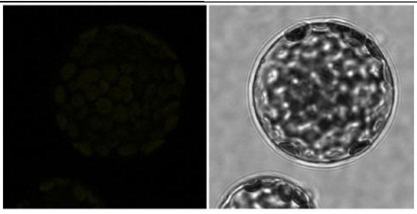
pSAT4A-<br>AtCBP20:cEYFP<br><br>and<br><br>pSAT1-nEYFP:AtSE	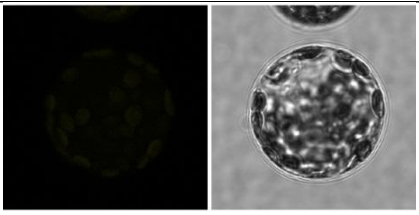

pSAT1-nEYFP:AtCBP80<br><br>and<br><br>pSAT4-cEYFP:AtSE	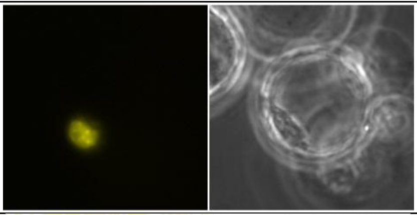
pSAT1-nEYFP:AtCBP80<br><br>and<br><br>pSAT4A-AtSE:cEYFP	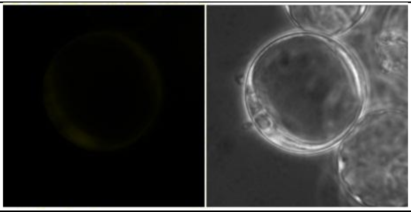
pSAT1A-<br>AtCBP80:nEYFP<br><br>and<br><br>pSAT4-cEYFP:AtSE	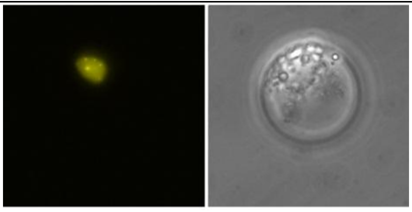
pSAT1A-<br>AtCBP80:nEYFP<br><br>and<br><br>pSAT4A-AtSE:cEYFP	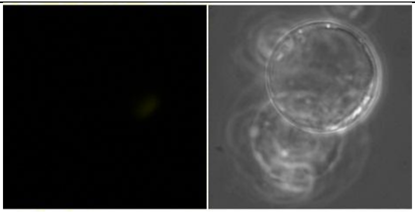
pSAT4-cEYFP:AtSE<br><br>and<br><br>pSAT1-nEYFP	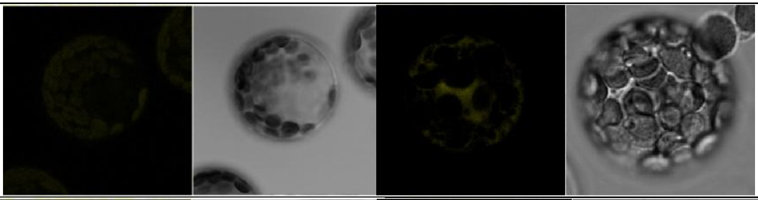
pSAT4A-AtSE:cEYFP<br><br>and<br><br>pSAT1-nEYFP	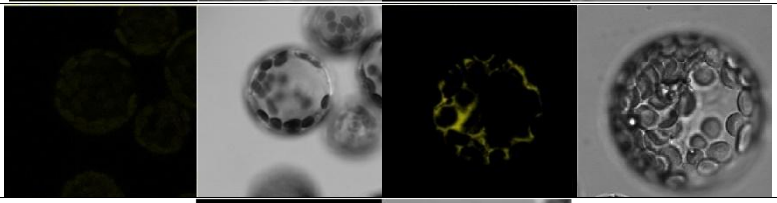
pSAT1-nEYFP:AtCBP20<br><br>and<br><br>pSAT4-cEYFP	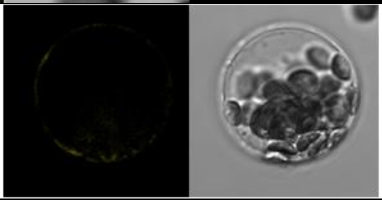
pSAT1A-<br>AtCBP20:nEYFP<br><br>and<br><br>pSAT4-cEYFP	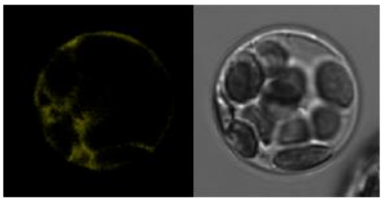
Multicassette vector<br>pPZP-RCS2:<br>- nEYFP:AtCBP80<br>- cEYFP<br>- mRFP	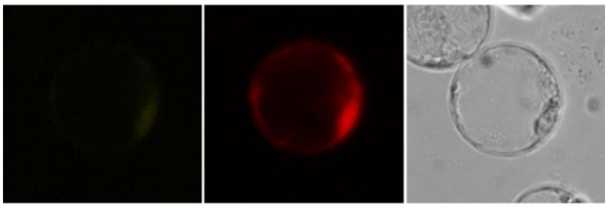

Multicassette vectorpPZP-RCS2:<br>-nEYFP<br>- cEYFP:AtCBP20<br>-mRFP	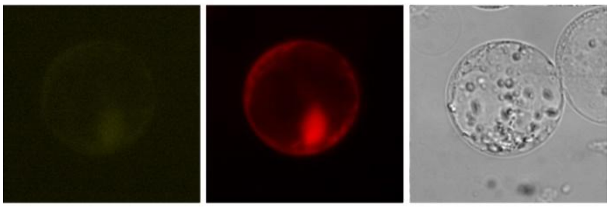
pSAT4-cEYFP:AtCBP20<br><br>and<br><br>pSAT1-nEYFP:AtCBP80	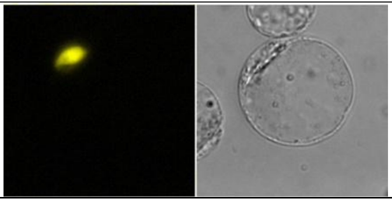
